# Editorial: Immunometabolic circuits in host-pathogen interaction

**DOI:** 10.3389/fcimb.2026.1870765

**Published:** 2026-06-02

**Authors:** Jaime David Acosta-España, Samuel Martins Gonçalves, Diana Santos-Ribeiro, Relber Aguiar Gonçales, Ricardo Silvestre, Rita Silva-Gomes

**Affiliations:** 1 Health Sciences Faculty, Universidad Internacional SEK (UISEK), Quito, Ecuador; 2 School of Medicine, Pontificia Universidad Católica del Ecuador, Quito, Ecuador; 3 Clínica Médica, Ribeirão Preto School of Medicine, University of São Paulo, São Paulo, Brazil; 4 Centro de Investigación para la Salud en América Latina (CISeAL), Pontificia Universidad Católica del Ecuador, Quito, Ecuador; 5 Life and Health Sciences Research Institute (ICVS), School of Medicine, University of Minho, Braga, Portugal; 6 ICVS/3B’s - PT Government Associate Laboratory, Braga/Guimarães, Portugal

**Keywords:** bacteria, CRP, IgA, immunometabolism, iron, macrophages, NK cell, virus

The mechanistic interplay between host immunity and pathogen survival is fundamentally governed by cellular bioenergetics (**Table 1**). Current evidence positions immune cell metabolism not merely as an auxiliary process to generate energy but as a central regulatory mechanism that determines immune cell fate and effector functions. The Research Topic “Immunometabolic Circuits in Host and Pathogen Interaction” presents seven pivotal studies that elucidate how metabolic networks, micronutrient trafficking, and interkingdom crosstalk operate across different biological scales to determine the clinical outcomes of infectious diseases.

**Table 1:** Metabolic vulnerabilities and host-directed therapeutic targets identified in the research topic.

Infection model / interface	Central immunometabolic shift	Key molecular / transcriptomic nodes	Proposed pathological consequence	Potential host-directed intervention	Reference
Lethal Influenza A (H1N1)	Systemic energy redistribution and oxidative stress	C-reactive protein (CRP)	Hyperinflammation and metabolic collapse	CRP-mediated systemic buffering	Luo et al. 2026
Severe COVID-19	NK cell metabolic exhaustion	HIF1A down, GLUT1 down, NFKB1 up	Impaired antiviral cytotoxicity	Restoration of glycolytic flux / Hypoxic adaptation	Osuna-Espinoza et al. 2025
Macrophage Bacterial Infection	Aerobic glycolysis and epigenetic modulation	PKM2, Lactate, Succinate, NAD+/NADH	Subversion of M1 microbicidal responses	Kinase network targeting (AMPK/mTOR)	Wang W-r. et al. 2026
Systemic Infection / Inflammation	Altered subcellular micronutrient trafficking	Lysosomal Iron, Mitochondrial respiration	Loss of nutritional immunity / Microbial proliferation	Iron sequestration modulation	Hoffmann et al. 2026
Chronic *M. tuberculosis*	Intracellular lipid retention	Liver X Receptors (LXR), Cholesterol	Enhanced intracellular mycobacterial survival	Pharmacological LXR activation (Cholesterol efflux)	Maceiras et al. 2025
Prokaryotic / Eukaryotic Interface	ATP-dependent post-translational subversion	AMPylators, Host cytoskeletal targets	Neutralization of innate immune signaling	Targeted AMPylation inhibitors	Wang X. et al. 2026
Respiratory Mucosal Axis	Dysbiosis of local immune education	Lung Microbiome, Secretory IgA (S-IgA)	Breakdown of barrier integrity / Chronic inflammation	Microbiome-targeted mucosal restoration	Stavart et al. 2026

Viral pathogens exert immense selective pressure on host cellular machinery, frequently inducing profound immunometabolic reprogramming to establish an intracellular niche conducive to replication. The pathophysiological trajectory of severe viral infections is heavily dictated by the host’s capacity to maintain bioenergetic homeostasis during systemic inflammation. Recent investigations have demonstrated that severe influenza A virus infection triggers massive energy redistribution and oxidative stress, which can be counterbalanced by specific host acute-phase proteins. Experimental models have revealed that C-reactive protein plays a critical immunometabolic role during severe H1N1 infection by restraining hyperinflammation and preventing fatal metabolic collapse (Luo et al.). This dynamic extends to SARS-CoV-2 pathogenesis, where the clinical severity of COVID-19 has been linked to metabolic exhaustion of the innate immune system. Patients with severe SARS-CoV-2 infection exhibit a hyperactivated, yet dysfunctional, natural killer cell phenotype, characterized by profound transcriptomic dysregulation (Osuna-Espinoza et al.). Specifically, the pronounced downregulation of key metabolic transcripts, including *HIF1A* and *GLUT1*, coupled with the upregulation of *NFKB1*, indicates a systemic failure in glycolytic adaptation that ultimately compromises antiviral immunity. Patient stratification further confirmed that an antagonist regulation of these factors correlates with adverse patient survival metrics.

Bacterial infections trigger equally complex bioenergetic shifts, primarily orchestrated through the metabolic rewiring of macrophages. The transition toward aerobic glycolysis during a bacterial challenge supplies rapid energy while simultaneously yielding critical intermediate metabolites that govern downstream inflammatory cascades. Wang et al. review how the enzymatic modulation of targets such as PKM2, alongside the accumulation of lactate, succinate, and itaconate, directly controls the NAD+/NADH redox balance (Wang et al.). These metabolic intermediates function as potent epigenetic modulators and signaling molecules that dictate macrophage polarization. The coordination of these immune responses relies on highly integrated kinase networks, including HIF-1α, NF-κB, PI3K/Akt/mTOR, and AMPK (Wang et al.). By exploiting specific host metabolic pathways, persistent bacterial pathogens can subvert pro-inflammatory microbicidal responses, underscoring the therapeutic necessity of targeting glycolytic reprogramming to circumvent antimicrobial resistance.

Beyond classical carbohydrate metabolism, the subcellular partitioning of trace elements and lipids serves as a crucial regulatory axis in host defense. Hoffmann et al. review the role of iron as a potent immunometabolic signal that orchestrates immune cell differentiation, mitochondrial respiration, and inflammatory feedback loops (Hoffmann et al.). The dynamic trafficking of iron within host lysosomes directly coordinates the restriction of microbial growth and prevents pathogen access to essential metalloenzymes, extending the bioenergetic landscape into the realm of nutritional immunity. In the context of chronic bacterial persistence, host lipid metabolism profoundly dictates disease severity. Transcriptomic analyses of whole blood from human cohorts and experimental murine models establish a definitive correlation between tuberculosis severity and the activation of liver X receptors (LXR) (Maceiras et al.). Pharmacological stimulation of the LXR pathway enhances critical cholesterol efflux mechanisms, thereby constraining the intracellular environment and improving the long-term bacterial control of *Mycobacterium tuberculosis* infection (Maceiras et al.).

Adding a further layer of sophistication to these metabolic interactions, specific post-translational modifications operate as bioenergetic switches during host-pathogen arms races, as surveyed by Wang et al. AMPylation utilizes cellular ATP to covalently transfer an AMP moiety to target proteins, thereby regulating critical biological functions ranging from bacterial redox homeostasis to eukaryotic endoplasmic reticulum stress responses (Wang et al.). Virulent pathogens frequently secrete specialized AMPylators to hijack host cytoskeletal dynamics and neutralize innate immune signaling, showcasing a direct exploitation of host nucleotide metabolism.

Finally, Stavart et al. delve into the continuous calibration of immunometabolic equilibrium at mucosal surfaces by interkingdom communication. The respiratory microbiome, comprising a diverse consortium of bacteria, fungi, viruses, and archaea, is essential for local immune education (Stavart et al.). Bidirectional crosstalk between these microbial communities and the mucosal secretory immunoglobulin A system preserves pulmonary barrier integrity. Dysregulation of this precise mucosal axis facilitates colonization by opportunistic pathogens and drives chronic inflammatory lung pathologies.

Collectively, the articles in this Research Topic underscore a unifying concept: immunometabolism operates as a multi-scale regulatory network that integrates intracellular signaling, tissue microenvironments, and systemic nutrient dynamics to shape host–pathogen interactions. This perspective transcends reductionist views of immune responses, highlighting the dynamic and interconnected nature of metabolic and immunological processes.

Looking forward, several challenges and opportunities remain. A deeper understanding of the temporal dynamics of metabolic reprogramming during infection, the integration of metabolic signals across different cell types and tissues, and the influence of host genetic and environmental factors will be essential.

In conclusion, this Research Topic provides a timely and integrative overview of how metabolic circuits govern host–pathogen interactions. By bridging molecular mechanisms with system-level insights, these studies pave the way for a more nuanced understanding of immunity and open new avenues for therapeutic intervention in infectious diseases.

